# Chemotherapy resistance and stemness in mitotically quiescent human breast cancer cells identified by fluorescent dye retention

**DOI:** 10.1007/s10585-018-9946-2

**Published:** 2018-10-30

**Authors:** Lewis A. Quayle, Penelope D. Ottewell, Ingunn Holen

**Affiliations:** Department of Oncology and Metabolism, Medical School, University of Sheffield, Beech Hill Road, Sheffield, S10 2RX UK

**Keywords:** Breast cancer, Stem cell, Dormancy, Quiescence

## Abstract

Metastatic recurrence in breast cancer is a major cause of mortality and often occurs many years after removal of the primary tumour. This process is driven by the reactivation of disseminated tumour cells that are characterised by mitotic quiescence and chemotherapeutic resistance. The ability to reliably isolate and characterise this cancer cell population is critical to enable development of novel therapeutic strategies for prevention of breast cancer recurrence. Here we describe the identification and characterisation of a sub-population of slow-cycling tumour cells in the MCF-7 and MDA-MB-231 human breast cancer cell lines based on their ability to retain the lipophilic fluorescent dye Vybrant^®^ DiD for up to six passages in culture. Vybrant^®^ DiD-retaining (DiD+) cells displayed significantly increased aldehyde dehydrogenase activity and exhibited significantly reduced sensitivity to chemotherapeutic agents compared to their rapidly dividing, Vybrant^®^ DiD-negative (DiD−) counterparts. In addition, DiD+ cells were exclusively capable of initiating population re-growth following withdrawal of chemotherapy. The DiD+ population displayed only partial overlap with the CD44^+^CD24^−/low^ cell surface protein marker signature widely used to identify breast cancer stem cells, but was enriched for CD44^+^CD24^+^ cells. Real-time qPCR profiling revealed differential expression of epithelial-to-mesenchymal transition and stemness genes between DiD+ and DiD− populations. This is the first demonstration that both MCF-7 and MDA-MB-231 human breast cancer lines contain a latent therapy-resistant population of slow-cycling cells capable of initiating population regrowth post-chemotherapy. Our data support that label-retaining cells can serve as a model for identification of molecular mechanisms driving tumour cell quiescence and de novo chemoresistance and that further characterisation of this prospective tumour-reinitiating population could yield novel therapeutic targets for elimination of the cells responsible for breast cancer recurrence.

**Electronic supplementary material:**

The online version of this article (10.1007/s10585-018-9946-2) contains supplementary material, which is available to authorized users.

## Introduction

Metastatic recurrence in advanced breast cancer is a major cause of patient morbidity and mortality. A substantial body of evidence indicates that metastatic foci are initiated by a latent sub-population of cancer cells that are capable of surviving adjuvant chemotherapy and colonising distal sites, where they may remain dormant for several years before emerging to reinitiate tumour growth [1]. The inability to remove this population, often referred to as cancer stem cells (CSCs), has proved to be the major obstacle in developing truly curative treatments for advanced breast cancer, with 15-year recurrence rates remaining as high as 40% [2].

Given that traditional chemotherapeutic agents are reliant on mitotic activity to initiate cytotoxicity, it has been proposed that CSCs, like their non-malignant counterparts, exist in a slow-cycling state of relative mitotic quiescence that confers an inherent ability to survive chemotherapy. Indeed, the slow-cycling status of adult stem cell populations identified in the brain, skin and intestine has been transitively linked to their observed survival during chemotherapy and their subsequent ability to regenerate tissue following chemotherapy withdrawal [3–5]. Analogous populations of slow-cycling cells have been identified in multiple cancer types. Yumoto et al. [6] described the identification of slowly cycling sub-populations in a number of human prostate cancer cell lines based on their persistent retention of fluorescent lipophilic dye. Expanding on these findings, Wang et al. [7] demonstrated that label-retaining prostate cancer cells differentially up-regulate expression of various haematopoietic stem cell niche-associated markers and were significantly more metastatic in vivo when compared to the rapidly dividing cell population isolated from the same parental culture. Dembinski and Krauss [8] similarly reported that a slow-cycling population of label-retaining pancreatic adenocarcinoma cells demonstrated morphological and transcriptomic changes indicative of epithelial-to-mesenchymal (EMT) transition and were ten-fold more tumourigenic in vivo than the non-label retaining population. Label-retaining populations have also been identified in melanoma, brain, ovarian, colon and breast cancer cell lines [9–12]. However, the link between these slow-cycling, label-retaining populations and chemoresistance has remained largely unstudied and it remains unclear whether label-retaining cells represent a tumour recurrence-initiating population.

In this study, we demonstrate the novel application of Vybrant^®^ DiD for identification, isolation and characterisation of a latent, slow-cycling, label-retaining cell population in the oestrogen receptor-positive MCF-7 and triple-negative MDA-MB-231 human breast cancer cell lines. We demonstrate that these slow-cycling cells are significantly more resistant to conventional chemotherapeutic agents than their rapidly dividing counterparts and importantly, that label-retaining cells are exclusively capable of active proliferation following removal of chemotherapeutic drugs, implying their ability to drive tumour recurrence. Our data support that label-retaining cells can serve as a model for identification of molecular mechanisms driving tumour cell quiescence and de novo chemoresistance.

## Materials and methods

### Cell culture

MCF-7 and MDA-MB-231 human breast cancer cell lines were sourced directly from the American Type Culture Collection (ATCC) (Manassas, Virginia, USA) as fully authenticated cryogenically frozen cultures. Cells were routinely maintained in vitro as adherent cultures grown in complete growth medium composed of RPMI-1640 basal medium (11 mM glucose, 2 mM L-glutamine) (Life Technologies Ltd., Paisley, U.K.) supplemented with 10% (v/v) foetal bovine serum (FBS) (Life Technologies Ltd.).

### Fluorescent dye labelling, label retention assays and fluorescence-activated cell sorting

Vybrant^®^ DiD labelling was performed according to the manufacturer’s instructions for labelling cells in suspension (Molecular Probes MP22885) using 1.0 × 10^6^ cells per ml of serum-free basal culture medium.

Labelled samples were grown as adherent cultures for up to six consecutive passages post-staining, with sub-culture being undertaken at approximately 80% of the duration of the logarithmic phase of culture growth. At each sub-culture interval, samples of 1.0 × 10^6^ cells were prepared in serum-free basal medium and used to assess the degree of Vybrant^®^ DiD staining present within each sample. Cytofluorimetric analyses were undertaken using the BD™ LSR-II™ platform (Beckton, Dickenson and Co. Plc., Oxford, UK). Fluorescence activated cell sorting (FACS) was undertaken using the BD™ FACSAria™ platform (Beckton, Dickenson and Co. Plc.) after six consecutive passages of culture post-labelling. Cytofluorimetric analysis and FACS platforms were calibrated at the outset of each experiment to enable detection of Vybrant^®^ DiD-negative (DiD-) and -positive (DiD+) cells. Negative control samples consisting of cells that had never been exposed to Vybrant^®^ DiD were first used to set the gate for the detection of DiD− events. All cells subsequently identified as DiD− had therefore completely lost their initial label and possessed a fluorescence intensity equivalent to that of an unlabelled cell population. Positive control samples consisting of cells that had been freshly labelled with Vybrant^®^ DiD were then used to set the gate for detection of DiD+ cells. All cells subsequently identified as DiD+ (dye-retaining) therefore possessed a fluorescence intensity equivalent to that of a dye-saturated cell population immediately post-labelling. The general gating tree for identification, differential flow cytometric analysis and isolation of DiD− and DiD+ cells is shown in Supplementary Fig. 1.

### Flow cytometric assays

In order to analyse the cell cycle profile of live DiD− and DiD+ cells, unsorted samples were prepared at 1.0 × 10^6^ cells per ml in serum-free basal culture medium from labelled cultures at six passages post-staining. Hoechst 33342 stock solution (Life Technologies Ltd.) was added to samples at a final concentration of 5 µg/ml and incubation undertaken at 37 °C for 45 min in total darkness prior to analysis.

Aldehyde dehydrogenase (ALDH) activity was determined using the non-immunological ALDEFLUOR™ assay kit (STEMCELL™ Technologies U.K. Ltd., Cambridge, U.K.) according to the manufacturer’s instructions (STEMCELL™ Technologies Document 29888) using unsorted cultures at six passages post-staining with Vybrant^®^ DiD. Samples of unlabelled cells treated with the specific ALDH inhibitor diethylaminobenzaldehyde (DEAB) were prepared in an identical manner concomitantly as control samples used to allow definition of the gating strategy and to control for autofluorescence by setting the appropriate photomultiplier tube voltage for detection of negative events within each channel.

In order to analyse the expression of breast CSC markers, unsorted Vybrant^®^ DiD-labelled samples at passage six post-labelling were stained with mouse monoclonal IgG1 anti-human CD24-phycoerythrin (Abcam Plc., Cambridge, U.K., clone SN3, product code ab77219) and mouse monoclonal IgG2b anti-human CD44-Brilliant Violet 421™ (Beckton, Dickenson and Co. Plc., clone G44-26, product code 562890) primary antibodies concomitantly. The final working concentration of each antibody was 6 µg/ml and 10 µg/ml, respectively. Matched isotype control samples were prepared using phycoerythrin-conjugated mouse monoclonal IgG1 (Abcam Plc., clone B11/6, product code ab91357) and Brilliant Violet 421™-conjugated mouse monoclonal IgG2b (Beckton, Dickenson and Co. Plc., clone 27–35, product code 562748) isotype control antibodies. Each isotype control antibody was used at the same working concentration as the primary antibody to which it was matched. Briefly, samples of 1.0 × 10^6^ live cells were prepared from harvested cell cultures and washed twice by resuspension in flow cytometry buffer (5% (v/v) FBS in PBS). Washed cell pellets were resuspended in 100 µl of primary or isotype control antibody solution (pre-diluted to the desired concentration in flow cytometry buffer) and incubated for 1 h at 4 °C in total darkness under constant agitation. Samples were then washed three times, resuspended in 1 ml of flow cytometry buffer and passed through a 40 µm cell strainer prior to immediate cytofluorimetric analysis.

### Immunofluorescence

Vybrant^®^ DiD− and DiD+ cells were sorted at passage six post-labelling of cultures and 2000 sorted cells deposited onto Superfrost Plus™ glass microscopy slides by cytocentrifugation (150x*g* for 3 min using medium acceleration) using the Shandon™ Cytospin™ 3 cytocentrifuge (Thermo Fisher Scientific, Paisley, UK). Samples were fixed in 4% (w/v) paraformaldehyde on ice for 10 min, washed in two changes of PBS, and permeabilised in 0.1% (v/v) Triton™ X-100 in PBS. Samples were washed three times using PBS-Tween^®^ 20 (PBST) (0.01% (v/v) Tween^®^ 20 in PBS) and blocked in a solution of 10% (v/v) normal goat serum + 1% (w/v) bovine serum albumin (BSA) in PBST at ambient temperature for 1 h. Immunostaining for Ki67 expression was undertaken using an unconjugated rabbit polyclonal IgG anti-human Ki67 primary antibody (Abcam Plc., product code ab15580) diluted in in 1% (w/v) BSA in PBST to a final working concentration of 1 µg/ml. Matched isotype control samples were prepared using an unconjugated rabbit polyclonal IgG isotype control antibody (Abcam Plc., product code ab171870) and were used at the same final working concentration as the primary antibody. Incubation was undertaken inside of a humidified slide tray overnight at 4 °C. Following three more washes in PBST, primary antibody staining was localised using an AlexaFluor^®^488-conjugated goat polyclonal anti-rabbit IgG secondary antibody (Abcam Plc., product code ab150077) diluted in in 1% (w/v) BSA in PBST to a final working concentration of 4 µg/ml. Incubation was undertaken for 1 h at ambient temperature. Three further washes in PBST were undertaken before samples were mounted using ProLong^®^ Gold anti-fade mounting medium containing 4ʹ,6-diamidino-2-phenylindole (DAPI) (Life Technologies Ltd.).

### Chemotherapy resistance assays

Cultures were labelled with Vybrant^®^ DiD and grown for five consecutive passages post-staining before being seeded into triplicate wells of six-well cluster plates. All plates were placed into incubation for a period of 24 h post-seeding prior to treatment with either pegylated liposomal doxorubicin (Clinical Research Pharmacy, Weston Park Hospital, Sheffield, U.K.) or paclitaxel (Abcam Plc.) at the pre-determined IC_95_ concentration (2.36 µM and 46.45 nM, respectively, for MCF-7 cells and 0.32 µM and 20.80 nM, respectively, for MDA-MB-231 cells). These concentrations were established using drug dose–response curves generated using the MTT assay (Supplementary Fig. 2) as previously described [13]. After 72 h, drug-containing medium was removed from cultures and Vybrant^®^ DiD content analysed cytofluorimetrically. Vybrant^®^ DiD− and DiD+ populations were sorted by FACS and deposited directly into triplicate wells of six-well plates containing drug-free culture medium at a clonogenic density of 200 cells per well. Plates were incubated for a period equivalent to at least six log-phase doubling times prior to colony formation being assayed. Colonies were fixed in a 4% (w/v) paraformaldehyde in PBS for 15 min at ambient temperature. Fixative was then removed and colonies stained with 0.05% (w/v) aqueous crystal violet solution (Merck Chemicals Ltd., Nottingham, U.K.) for 30 min at ambient temperature. Plates were imaged using the Pixera Professional 1.2 megapixel digital camera system (Pixera UK Ltd., Bourne End, UK) and colonies counted by way of semi-automated image analysis using GeneTools software (Syngene U.K. Ltd., Cambridge, UK).

### Real-time qPCR

Total RNA was isolated from DiD− and DiD+ cells at passage six post-labelling of cultures using the miRNeasy Micro Kit (Qiagen UK, Manchester, UK) according to the manufacturer’s instructions. During FACS, each population was sorted directly into separate tubes containing 700 µl of QIAzol^®^ lysis reagent. Following extraction of RNA, cDNA was synthesised using the RT^2^ First Strand Kit (Qiagen) and real-time qPCR carried out using RT^2^ SYBR Green ROX™ qPCR Mastermix (Qiagen) according to the manufacturer’s instructions. The complete PCR reaction mixture (Mastermix + cDNA synthesis product) was dispensed as 10 µl volumes into the wells of a 384-well RT^2^ custom PCR array containing pre-dispensed primers (information for primers is available in Supplementary Table 1). A total of 1.25 ng of cDNA was used per 10 µl reaction. The real-time qPCR amplification protocol was run on an Applied Biosystems 7900-HT real-time cycler and was as follows: 95 °C for 10 min (1 cycle) followed by 15 s at 95 °C and 1 min at 60 °C (40 cycles). Dissociation (melting) curve analysis was carried out at the end of each run using the default melting curve programme of the cycler.

### Statistical analyses

All data are expressed as the mean ± the standard error of the mean (SEM). All statistical analyses were undertaken using GraphPad Prism 7 (GraphPad Software Inc., La Jolla, USA). The statistical analysis applied to data, where undertaken, is indicated within the respective figure legend. Statistical significance was attributed when P < 0.05.

## Results

### Vybrant^®^ DiD is well suited to long-term lineage tracing

The lipid intercalating long alkyl side-chain carbocyanine derivatives, such as the Vybrant^®^ and structurally analogous PKH dye series, are by far the most frequently used supravital lipophilic fluorochromes for cell tracing and tracking applications in cancer studies [14, 15]. When present within biological membranes, dyes of both series characteristically exhibit strong fluorescence and photostability as a result of their extremely high extinction coefficients, modest quantum yields, and short excited-state lifetimes within lipid environments, making their lipid intercalating and photochemical properties ideally suited to long-term lineage tracing applications [16, 17]. However, the potential for confounding results due to inadvertent lateral dye transfer, cytotoxicity and loss of cellular function following initial staining is greater when using PKH rather than Vybrant^®^ dyes based on literary reports [15, 18]. In addition, the initial labelling of cells with PKH dyes requires resuspension of cells in an isosmotic mannitol-based loading medium while the Vybrant^®^ dyes can be added directly to culture medium, making handling less complicated and permitting uniform labelling of cells either in suspension or when growing as adherent monolayers [19]. Vybrant^®^ DiD was chosen for further assessment of suitability for lineage tracing from the Vybrant^®^ dyes commonly used for such experiments (Vybrant^®^ DiO, DiI, CM-DiI and DiD) based on preliminary experiments showing that none of these dyes adversely affected cell culture growth, colony formation or migratory capability, but that Vybrant^®^ DiD resulted in the most intensely fluorescent staining (data available upon request).

Initial experiments designed to assess the efficiency of cellular Vybrant^®^ DiD uptake and its effects on tumour cell viability were carried out using the highest supplier-recommended concentration of 5 µM. The percentage of positively labelled and viable cells in MCF-7 and MDA-MB-231 cultures was determined cytofluorimetrically immediately after DiD staining. Both MCF-7 and MDA-MB-231 cultures were 100% DiD positive (Supplementary Fig. 3a) and there was no significant difference in culture viability between unstained and DiD-stained cells in either cell line, as determined by cytofluorimetric assessment of propidium iodide uptake (Supplementary Fig. 3b). Next, the effects of DiD staining on the proliferation of MCF-7 and MDA-MB-231 cell lines was assessed by haemocytometric counting of viable cell number in unstained and DiD-stained cultures at 24-h intervals; no significant differences were detected at any time point in either cell line, confirming that the cell labelling protocol had no adverse effects on cell viability and growth (Supplementary Fig. 3c).

As the identification of cells with reduced mitotic activity by label-retention assays is critically dependent on dilution of fluorescent dye from actively proliferating cells, the degree of fluorescence in a labelled culture must negatively correlate with culture expansion. In order to determine the effects of cell proliferation on DiD intensity, the fluorescent signal of DiD-stained MCF-7 and MDA-MB-231 cultures was measured immediately post-staining and then again at the end of the logarithmic phase of one passage of culture growth (4 days in both instances). The resultant data confirmed that as the number of cells within each culture expanded, there was a reciprocal reduction in the intensity of DiD fluorescence (Supplementary Fig. 3d), demonstrating that the retention of DiD inversely correlates with net mitotic activity.

### Human breast cancer cultures contain a label-retaining sub-population

**Fig. 1:**
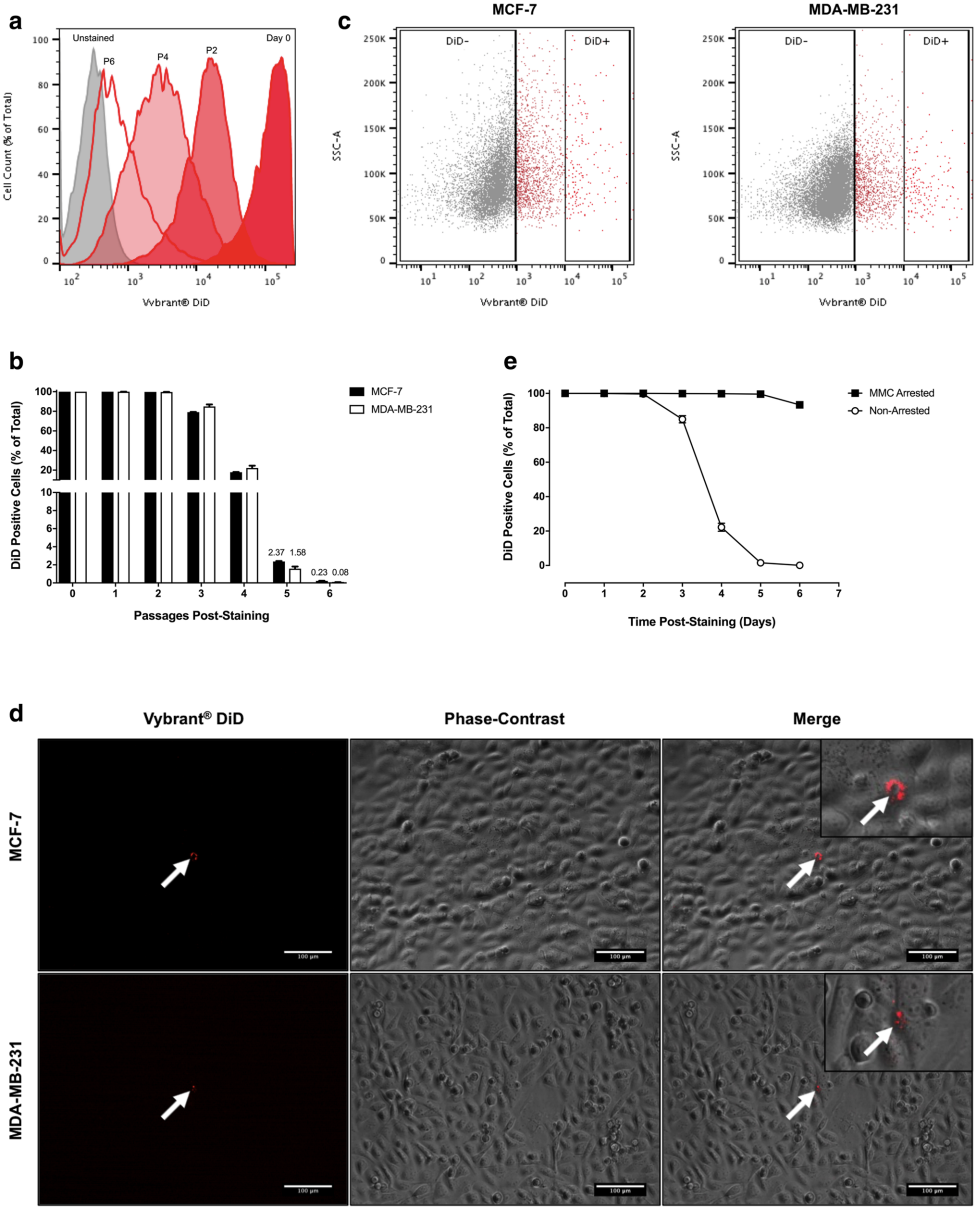
Identification of Label-Retaining Cells In Vitro. **a** Cytofluorimetric histograms illustrating dilution of Vybrant^®^ DiD in adherent human breast cancer cell cultures at passages 2, 4 and 6 of culture growth (P2, P4 and P6, respectively, where P = passage number). All cells were intensely positive immediately after staining (Day 0). Fluorescent dye was lost from rapidly proliferating cells to reveal a population of more slowly cycling label-retaining cells beyond passage 4. **b** Representative flow cytometry plots depicting Vybrant^®^ DiD negative (DiD−) and positive (DiD+) populations in adherent MCF-7 and MDA-MB-231 cultures at passage six post-staining. **c** Cytofluorimetrically determined percentage of Vybrant^®^ DiD-positive cells within adherent MCF-7 and MDA-MB-231 cultures over six consecutive passages of culture growth (n = 3). **d** Analysis of Vybrant^®^ DiD retention in MDA-MB-231 cultures in the presence and absence of mitomycin C (MMC) over six consecutive passages of culture growth (n = 3). **e** Fluorescent and phase-contrast image overlay of MCF-7 and MDA-MB-231 cultures at passage five post-staining (scale bar = 100 µm). White arrows indicate DiD+ cells (red). All graphical data are expressed as mean ± SEM

In order to determine whether DiD was progressively diluted from cultures over subsequent passages of growth, MCF-7 and MDA-MB-231 cells were stained with DiD and the proportion of dye-retaining cells analysed by flow cytometry at the point of sub-culture over six consecutive passages. As was previously observed, 100% of cells were initially positive for DiD (Fig. 1a, b). After three passages in culture, the mean number of dye-stained cells began to decrease (Fig. 1b). After five passages, the mean number of DiD− cells had exceeded 50%, while DiD+ cells accounted for 2.37 ± 0.05% of the total cell population in MCF-7 cultures and 1.56 ± 0.24% in MDA-MB-231 cultures (Fig. 1b, c). At this point, dye-retaining DiD+ cells were easily distinguishable from those cells that had lost their initial DiD label or those that retained only a low level of DiD staining by both flow cytometry and fluorescence microscopy (Fig. 1c, d).

A separate series of validation experiments were undertaken to confirm that the decrease in the number of dye-retaining cells was due to division (and conversely that retention was due to reduced mitotic activity); cells were growth-arrested by pre-treatment with mitomycin C (10 µg/ml for 3 h) prior to Vybrant^®^ DiD-labelling and the number of dye-retaining cells in both growing and growth-arrested cultures measured over time. No significant reduction in the number of dye-stained cells in mitomycin C-treated cultures were observed over the entire assay period, while the number of dye-retaining cells in growing cultures was reduced to ≤ 0.1% after six passages, in accordance with previous observations (Fig. 1e).

### Label-retaining MCF-7 and MDA-MB-231 cells are slow-cycling

**Fig. 2:**
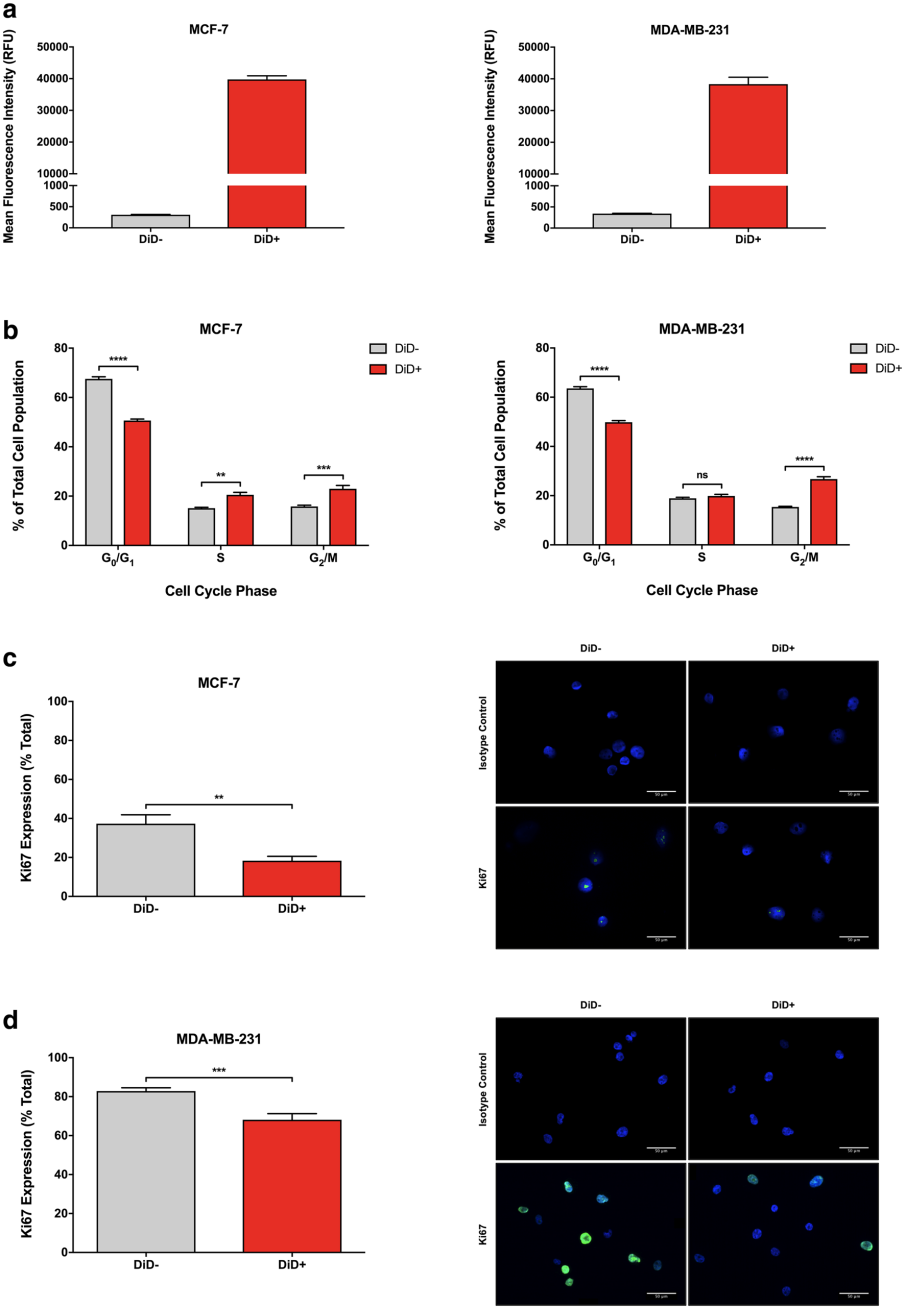
Label-retaining cells are slow-cycling. **a** Cytofluorimetrically determined mean fluorescence intensity (RFU = relative fluorescence units) of Vybrant^®^ DiD-negative (DiD−) and DiD-positive (DiD+) MCF-7 and MDA-MB-231 populations at passage 6 post-staining (n = 3). **b** Univariant cell cycle distribution of DiD− and DiD+ MCF-7 and MDA-MB-231 cells at 6 passages post-staining are shown; the percentage of cells within each population that are in G_0_/G_1_-phase (haploid chromosome number), S-phase (intermediate chromosome number) and G_2_/M-phase (diploid chromosome number) of the cell cycle are depicted (n = 3, two-way ANOVA with Sidak’s multiple comparison, ns = not statistically significant or P > 0.05, **** = P ≤ 0.0001). **c** Relative expression of Ki67 by DiD− and DiD+ MCF-7 cells (n = 3, unpaired *t*-test, ** = P ≤ 0.01). Representative images of immunostained cytocentrifuge preparations of DiD− and DiD+ MCF-7 cells isolated by FACS at passage 6 post-staining are shown; Ki67 staining (green) and nuclear counter-staining with DAPI (blue) are depicted (scale bar = 50 µm). **d** Relative expression of Ki67 by DiD− and DiD+ MDA-MB-231 cells (n = 3, unpaired *t*-test, *** = P ≤ 0.001). Representative images of immunostained cytocentrifuge preparations of DiD− and DiD+ MDA-MB-231 cells isolated by FACS at passage 6 post-staining are shown; Ki67 staining (green) and nuclear counter-staining with DAPI (blue) are depicted (scale bar = 50 µm). All graphical data are expressed as mean ± SEM

The mean fluorescence intensity of DiD− (mean fluorescence = 309.3 ± 9.7 RFU for MCF-7 and 341.7 ± 8.67 RFU for MDA-MB-231) and DiD+ (mean fluorescence = 39,766 ± 1143 RFU for MCF-7 and 38,315 ± 2190 RFU for MDA-MB-231) cell populations after six consecutive passages was used to calculate the cell cycle time of DiD+ cells relative to DiD− cells (Fig. 2a). The DiD+ population was determined to lag behind the DiD− population by ~ 7 divisions in both cell lines. Subsequent live cell cycle analysis in the MCF-7 cell line revealed a significant 5.43% and 7.13% increase in the number of DiD+ cells in the S- and G_2_/M-phases of the cell cycle, respectively, compared to their DiD− counterparts. A significant 16.90% reduction on the G_0_/G_1_ fraction in DiD+ MCF-7 cells was also observed (Fig. 2b). Analysis of Ki67 immunostaining revealed a significant 49.10% decrease in the expression of Ki67 in the DiD+ population compared to the DiD− population (Fig. 2c). Similarly, live cell cycle analysis in the MDA-MB-231 cell line showed a significant 11.33% increase in the number of DiD+ cells in the G_2_/M-phase of the cell cycle compared to their DiD− counterparts, with a corresponding 13.73% reduction in the G_0_/G_1_ fraction (Fig. 2b). In addition, analysis of Ki67 immunostaining demonstrated a significant 14.74% reduction in the expression of Ki67 in DiD+ MDA-MB-231 cells compared with the DiD− MDA-MB-231 population (Fig. 2d). Collectively, these findings suggest that the relatively slow-cycling status of the DiD+ population is the net result of a greater number of G_0_-arrested cells accompanied by extended G_2_/M-phase transition or arrest.

### Chemotherapy positively selects for repopulating label-retaining cells

**Fig. 3:**
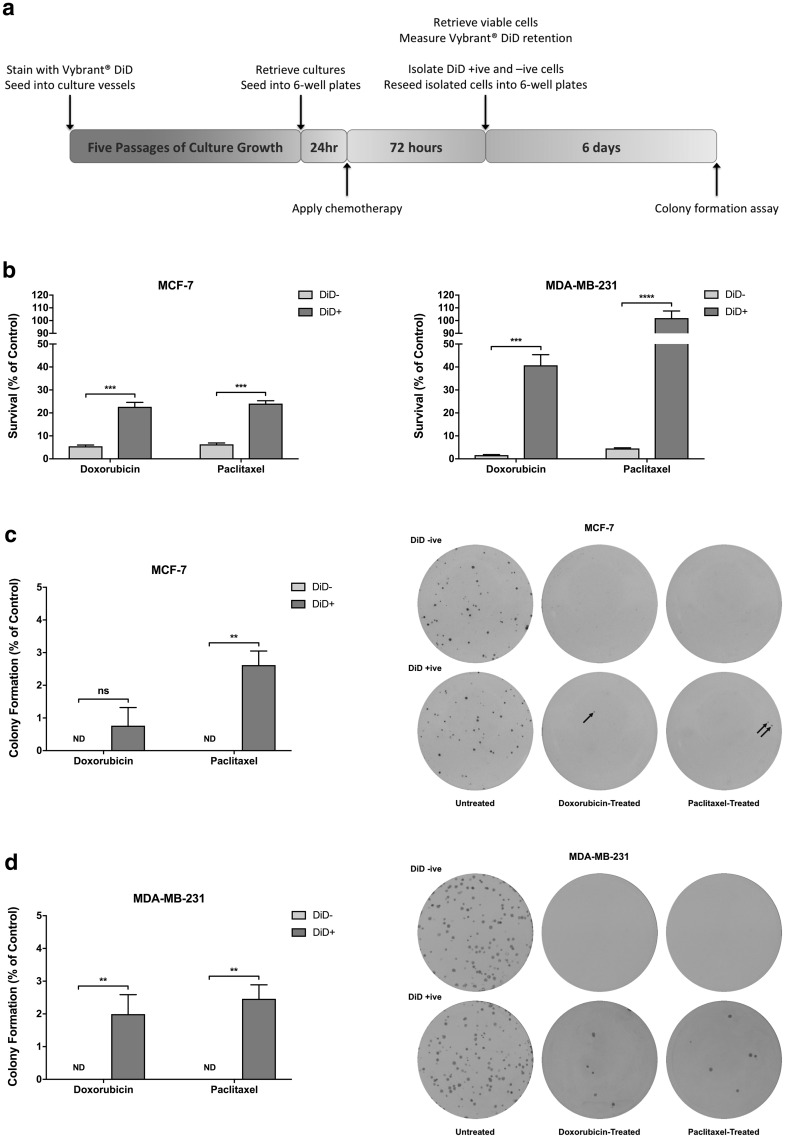
Label-retaining cells are resistant to chemotherapy. **a** Timeline for in vitro determination of chemotherapy resistance in Vybrant^®^ DiD-negative (DiD−) and Vybrant^®^ DiD-positive (DiD+) sub-populations. **b** Survival of DiD− and DiD+ MCF-7 and MDA-MB-231 cell populations following 72-h continuous treatment with the IC_95_ concentration of either doxorubicin or paclitaxel (n = 3, unpaired *t*-test, *** = P ≤ 0.001, **** = P ≤ 0.0001). **c** Colony formation by doxorubicin- or paclitaxel-treated DiD− and DiD+ cells isolated from MCF-7 cultures following cessation of treatment (n = 3, unpaired *t*-test, ND = not detected, ns = not statistically significant or P > 0.05, ** = P ≤ 0.01). Representative images of colony formation assays are also depicted. **d** Colony formation by doxorubicin- or paclitaxel-treated DiD− and DiD+ cells isolated from MDA-MB-231 cultures following cessation of treatment (n = 3, unpaired *t*-test, ND = not detected, ** = P ≤ 0.01). Representative images of colony formation assays are also depicted. All graphical data are expressed as mean ± SEM

Slow-cycling cells would be predicted to be resistant to the effects of standard chemotherapies. In order to determine whether exposure to high doses of commonly used chemotherapeutic drugs would enrich for DiD+ cells due to their slow-cycling nature, cultures of MCF-7 and MDA-MB-231 cells at passage five post-staining were treated with the pre-established IC_95_ concentration of either doxorubicin or paclitaxel (see experimental outline in Fig. 3a). After one further passage in culture, vehicle treated control MCF-7 cultures had expanded approximately two-fold while MDA-MB-231 control cultures had expanded approximately four-fold (data not shown). As expected, drug-treated cultures contained only a fraction of the total viable cell number present in the respective vehicle control (doxorubicin: MCF-7 = 5.51%, MDA-MB-231 = 1.88%; paclitaxel: MCF-7 = 6.40%, MDA-MB-231 = 5.18%). Upon analysis for Vybrant^®^ DiD staining, vehicle control-treated samples demonstrated the expected decrease in DiD+ cell content (reduced from 2.00 to 0.13% for MCF-7 and from 2.60 to 0.65% for MDA-MB-231) due to net dye dilution as the cultures continue to proliferate. In contrast, doxorubicin-treated (MCF-7 = 4.10-fold, MDA-MB-231 = 21.65-fold) and paclitaxel-treated (MCF-7 = 3.77-fold, MDA-MB-231 = 19.67-fold) samples demonstrated significant enrichment in DiD+ cell content (Fig. 3b). Collectively, these data demonstrate that slow-cycling DiD+ cells are less susceptible to chemotherapy-induced cytotoxicity than the rapidly-dividing DiD− cell population.

While the intrinsic ability of cancer cell populations to survive anti-neoplastic chemotherapy is clinically important, those cells that are able to initiate disease relapse or recurrence must also be capable of subsequent proliferation. In order to establish whether cells that survived exposure to chemotherapy were able to divide and establish clonal populations following cessation of treatment, DiD− and DiD+ populations were isolated from vehicle control-, doxorubicin- and paclitaxel-treated cultures by FACS and re-plated at clonal density in drug-free growth medium (see experimental outline in Fig. 3a). After seven days, there was no significant difference in the colony forming ability of the DiD− and DiD+ fractions in untreated (control) MCF-7 and MDA-MB-231 cultures (data not shown). Strikingly, a small proportion of DiD+ cells that had previously been exposed to either doxorubicin (MCF-7 = 0.76 ± 0.56%, MDA-MB-231 = 1.99 ± 0.59%) or paclitaxel (MCF-7 = 2.62 ± 0.43%, MDA-MB-231 = 2.46 ± 0.43%) formed new colonies after 7 days following removal of drugs, whereas no colonies were formed by DiD− cells isolated from either cell line post-treatment (Fig. 3c, d). When taken together, these data indicate that the slow-cycling status of the DiD+ cell population is associated with enhanced resistance to conventional chemotherapeutic agents, and importantly that these cells are exclusively responsible for re-establishing the tumour cell population following cessation of treatment, mimicking tumour relapse.

### Label-retaining cells differentially express putative breast cancer stem cell markers

Putative cancer stem cells have been shown to contribute to chemoresistance in a number of cancer types [20–23]. Given the established ubiquitous association of stemness and quiescence in the normal and neoplastic cellular hierarchies and the slow-cycling and therapy-resistant nature of the DiD+ cell population, we next investigated whether DiD-retaining cells displayed features of the purported breast cancer stem cell population.

**Fig. 4:**
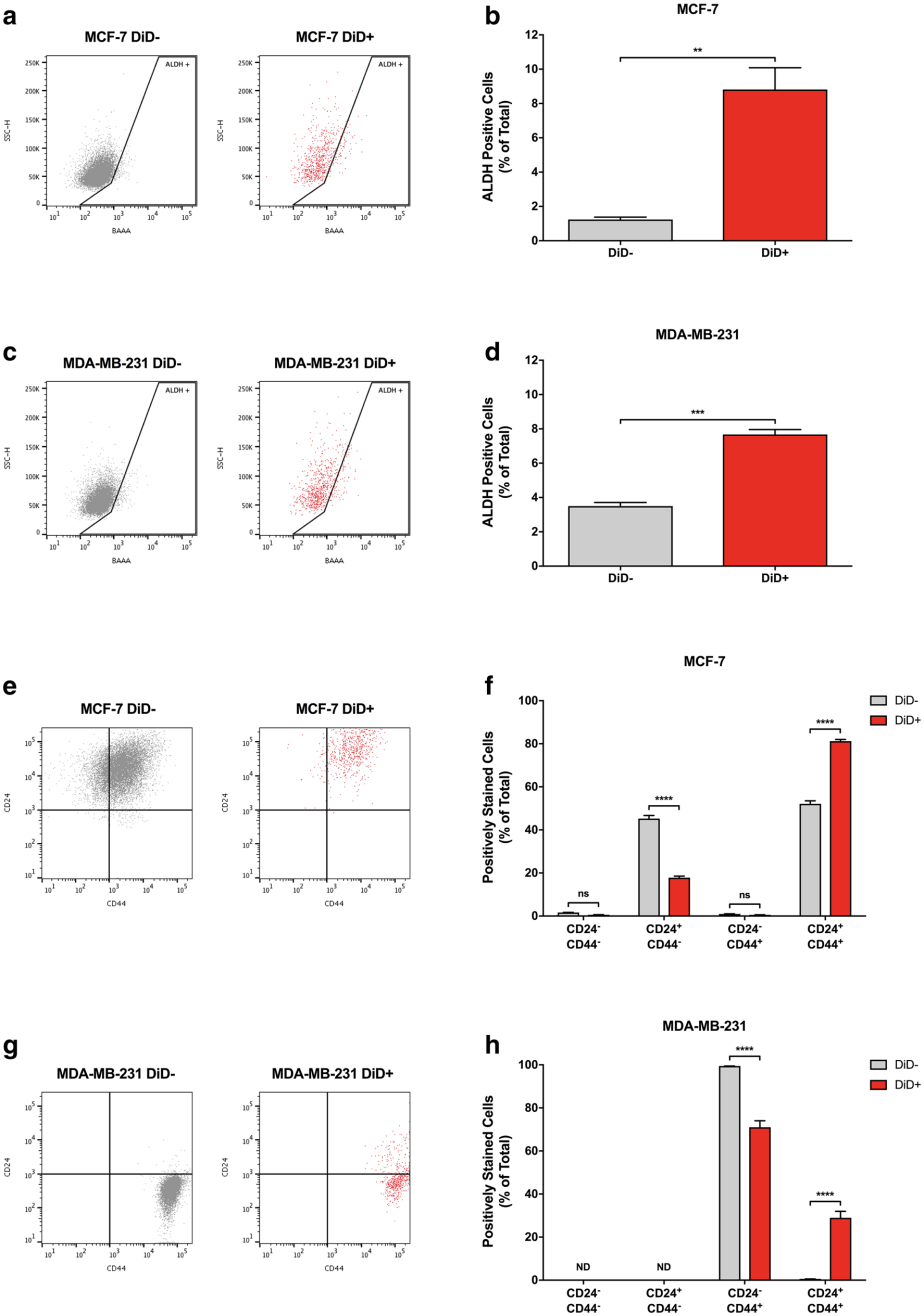
Label-retaining cells differentially express cancer stem cell markers. **a** Representative flow cytometry plots depicting aldehyde dehydrogenase (ALDH) activity in DiD− and DiD+ MCF-7 sub-populations. **b** Relative expression of ALDH by DiD− and DiD+ MCF-7 cells (n = 3, unpaired *t*-test, ** = P ≤ 0.01). **c** Representative flow cytometry plots depicting ALDH activity in DiD− and DiD+ MDA-MB-231 sub-populations. **d** Relative expression of ALDH by DiD− and DiD+ MDA-MB-231 cells (n = 3, unpaired *t*-test, *** = P ≤ 0.001). **e** Representative flow cytometry plots depicting CD24 and CD44 expression status of DiD− and DiD+ MCF-7 sub-populations. **f** Relative expression of CD24 and CD44 by DiD− and DiD+ MCF-7 cells (n = 3, two-way ANOVA with Sidak’s multiple comparison, ns = P > 0.05, **** = P ≤ 0.0001). **g** Representative flow cytometry plots depicting CD24 and CD44 expression status of Vybrant^®^ DiD− and DiD+ MDA-MB-231 sub-populations. **h** Relative expression of CD24 and CD44 by DiD− and DiD+ MDA-MB-231 cells (n = 3, two-way ANOVA with Sidak’s multiple comparison, ND = not detected, **** = P ≤ 0.0001). All graphical data are expressed as mean ± SEM

ALDH has been successfully used as a marker for isolation of stem-like cells from non-malignant tissue and multiple cancer types. In particular, seminal studies by Ginestier et al. [24] and Charafe-Jauffret et al. [25] have demonstrated the utility of ALDH activity for isolation of stem cell-like populations from normal human breast tissue and breast carcinomas. We therefore measured the intrinsic ALDH activity of DiD− and DiD+ populations cytofluorimetrically using the ALDEFLUOR™ assay. As shown in Fig. 4a, b, only 1.23 ± 0.14% of DiD− MCF-7 cells were ALDH^+^, while this was increased (7.16-fold) to 8.81 ± 1.27% in the DiD+ population. Similarly, 3.50 ± 0.21% of DiD− MDA-MB-231 cells were ALDH^+^, while this was increased (2.19-fold) to 7.68 ± 0.29% in the DiD+ population (Fig. 4c, d). These results demonstrate an overlap between the already described ALDH^+^ breast cancer sub-population and the DiD+ population identified here.

Along with ALDH, the CD44^+^CD24^−/low^ signature has also been widely used to demark a more tumourigenic population of supposed breast CSCs since it was first described by Al-Hajj et al. [26]. The co-expression of CD44 and CD24 within DiD− and DiD+ populations was therefore examined. In the DiD− MCF-7 cell population, 0.95 ± 0.08% of cells exhibited the CSC-associated CD44^+^CD24^−/low^ phenotype, with the remainder of cells being distributed between CD44^−^CD24^−/low^ (1.64 ± 0.05%), CD44^−^CD24^+^ (45.27 ± 1.44%) and CD44^+^CD24^+^ (52.13 ± 1.39%) phenotypes (Fig. 4e, f). Notably, these fractions were not significantly different in magnitude when compared to the relative size of each population measured in the parental MCF-7 cell population (data not shown). Interestingly however, while the size of the CSC-like CD44^+^CD24^−/low^ and CD44^−^CD24^−/low^ fractions remained unaltered when marker expression was measured in the DiD+ population, there was a significant 2.54-fold reduction in the CD44^−^CD24^+^ population and corresponding 1.56-fold increase in the CD44^+^CD24^+^ population (Fig. 4f). Moreover, almost the entire DiD− MDA-MB-231 population (99.45 ± 0.009%) was CD44^+^CD24^−/low^ while the remainder of cells (0.55 ± 0.009%) were CD44^+^CD24^+^ (Fig. 4g, h), again showing no significant difference with the proportional distribution of these markers measured in the parental cell line (data not shown). However, when the DiD+ population was analysed, a 1.40-fold reduction in CD44^+^CD24^−/low^ cells was measured, with a corresponding increase (52.41-fold) in the CD44^+^CD24^+^ population (Fig. 4h). When taken together, these data show that although a degree of overlap between the previously identified CSC markers and slow-cycling cells does exist, the slow-cycling label-retaining cell population are a distinct population of stem-like cancer cells.

### Label-retaining cells differentially express EMT and stemness genes

**Fig. 5:**
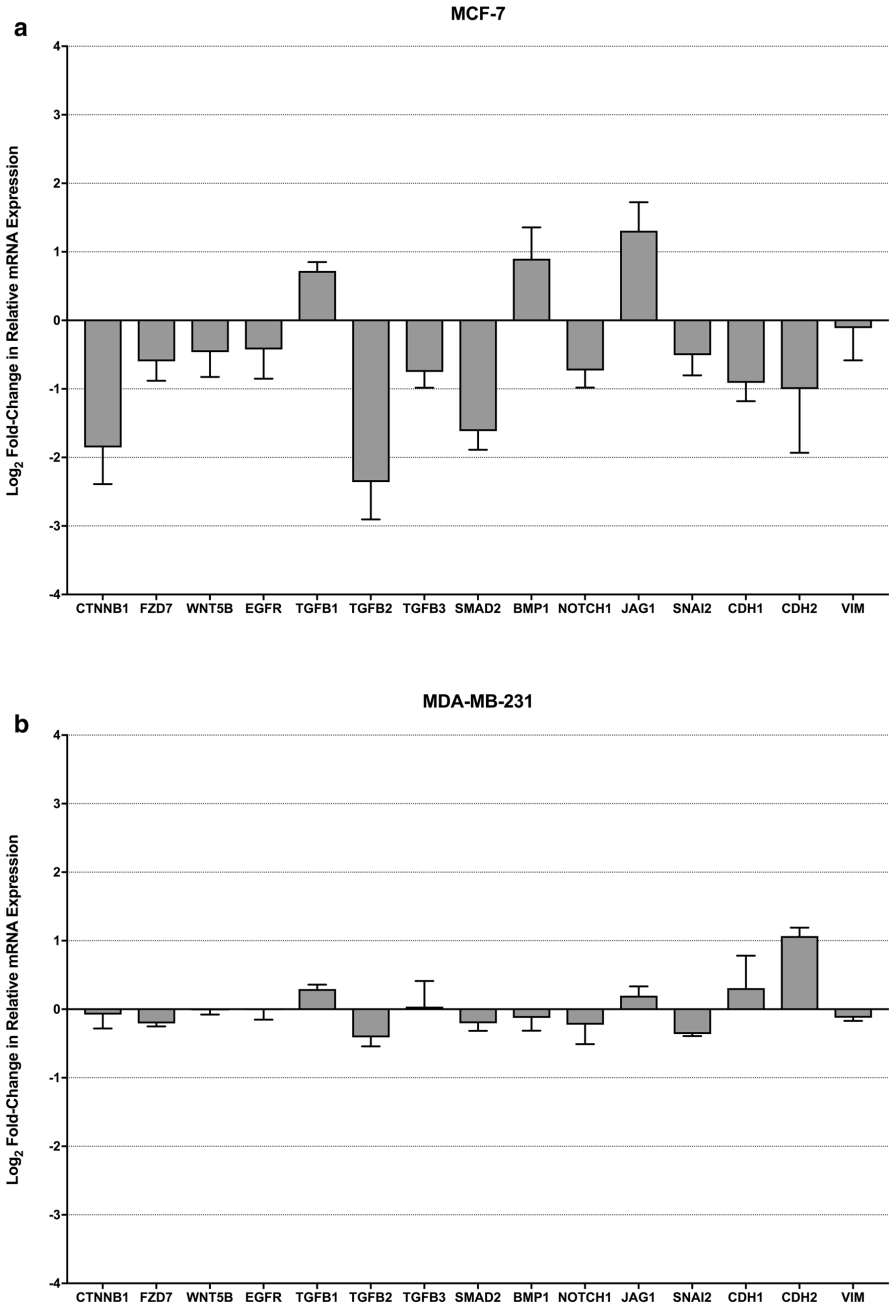
Label-retaining cells differentially express EMT and stemness genes. **a** Change in expression of genes associated with WNT-, TGFβ/BMP-, Notch-, EMT/core stemness-signalling pathways between DiD− and DiD+ MCF-7 sub-populations (n = 3). **b** Change in expression of genes associated with WNT-, TGFβ/BMP-, Notch-, EMT/core stemness-signalling pathways between DiD− and DiD+ MDA-MB-231 sub-populations (n = 3). Data are expressed as mean ± SEM

Given the chemoresistant properties of the DiD+ population and that they express markers associated with both mesenchymal and epithelial stemness, as has recently been associated with a more chemoresistant intermediate stem-like phenotype (reviewed by Fabregat et al. [27]), expression of genes relating to EMT were investigated by quantitative real-time PCR profiling of the DiD+ population relative to the DiD− population. As can be seen in Fig. 5a, while the expression of SNAI2 and CDH1 by DiD+ MCF-7 cells was decreased relative to DiD− cells, as would be expected of the EMT-associated cadherin-switch [28], the corresponding increase in CDH2 and VIM expression that would classically be associated with complete EMT were not evident. Moreover, EGFR and WNT-signalling genes (CTNNB1, FZD7 and WNT5B) that have known roles in regulation of EMT were also modestly down-regulated relative to the DiD− MCF-7 population. Interestingly, while components of TGFβ/BMP- (TGFB2/3 and SMAD2) and Notch-signalling (NOTCH1) were also down-regulated, three genes with known roles in functional differentiation (TGFB1, BMP1 and JAG1) were found to be up-regulated. By contrast, expression of all genes measured remained largely stable between DiD− and DiD+ MDA-MB-231 populations, with the exception of up-regulation of CDH2 in DiD+ MDA-MB-231 cells (Fig. 5b). Overall, these data indicate that slow-cycling DiD+ MCF-7 cells may not have undergone complete EMT, while slow-cycling DiD+ MDA-MB-231 cells largely share the transcriptomic profile of the already mesenchymal-like parental cell line.

## Discussion

In this report we have demonstrated that Vybrant^®^ DiD can be used for pulse-chase identification and characterisation of an intrinsic sub-population of slow-cycling cells in breast cancer cell lines based on their ability to retain their initial Vybrant^®^ DiD label for an extended duration. Compared to the rapidly dividing bulk cell mass, the reduced mitotic capacity of the label-retaining sub-population was shown to be associated with an array of biologically and clinically relevant differential characteristics. These included alterations in the expression of CD44 and CD24 surface markers, increased ALDH activity, transcriptomic profiles indicative of an intermediate EMT-MET phenotype, a marked reduction in chemosensitivity and an exclusive capacity for re-initiation of culture growth following cessation of exposure to chemotherapeutic agents. Collectively, these traits could signify that label-retaining cells are a population of cancer stem cells.

A number of previous studies have reported a significant expansion of the supposed cancer stem cell compartment following chemotherapy [8, 20–23]. Such enrichment would require either a large-scale expansion of the cancer stem cell population, or effective reduction of the bulk cell mass. A similar pattern of preferential proliferation or cytotoxicity would also be required to account for the significant increase in the relative proportion of slow-cycling, DiD+ cells surviving following exposure to chemotherapy agents reported here. Calculation of the absolute number of DiD+ cells present in MCF-7 cultures at the time of seeding and following exposure to a high concentration (IC_95_) of chemotherapeutic agents demonstrated an increase in the absolute label-retaining DiD+ cell number from ~ 1000 cells at the time of seeding to ~ 3600 cells at the end of the assay period. Similarly, in the MDA-MB-231 cell line the number of label-retaining DiD+ cells increased from ~ 1600 to ~ 2800 across all treatment groups. These increases in label-retaining cell numbers most likely occurred due to mitotic division generating partially labelled daughter cells. However, such expansion alone could not account for the significantly increased relative proportion of DiD+ cells within each of the drug-treated cultures compared to untreated cultures. These data not only demonstrate that label-retaining cells continued to proliferate when exposed to standard chemotherapeutic agents, but that the non-label-retaining DiD− cell population was significantly more susceptible to drug-induced cell death than their slow-cycling DiD+ counterparts, resulting in a net enrichment for DiD+ cells. These conclusions mirror those drawn from similar in vitro studies reported by Moore et al. [11], who also demonstrated effective enrichment of a slow-cycling fluorescent label-retaining population in vivo using clinically relevant doses of oxaliplatin and fluorouracil to treat tumours derived from the HCT116 human colon cancer cell line.

While the intrinsic ability of cancer cell populations to survive anti-neoplastic chemotherapy is clinically important, cells that are able to initiate tumoural relapse or disease recurrence must also be capable of subsequent proliferation. We therefore established that the DiD+ population were not avoiding the cytotoxic effects of doxorubicin or paclitaxel by entering a permanently non-dividing state of senescence, or that the onset of the cytotoxicity exerted by these drugs was simply delayed due to the slow-cycling nature of the label-retaining population. The formation of new clonal populations by label-retaining cells following cessation of exposure to chemotherapeutic drugs indicated that a proportion of this sub-population was completely resistant to chemotherapy. These findings mirror those previously reported by Moore et al. [11], which showed that a sub-population of label-retaining cells could actively proliferate in vitro and in vivo shortly after halting oxaliplatin and fluorouracil treatment. Moreover, the proportion of label-retaining cells that were able to form clonal populations following withdrawal of chemotherapy in this study (~ 2% for both MCF-7 and MDA-MB-231 across both chemotherapy drugs used) was several orders of magnitude larger than the proportion of cells previously demonstrated as being required to successfully establish metastasis (0.001–0.02%) [29–31]. When taken together, these results indicate that slow-cycling cells not only survive during drug exposure but also are capable of reactivation after withdrawal of chemotherapy and could therefore potentially initiate either local tumoural relapse or formation of a secondary metastatic lesion.

Comparative live cell cycle profiling of DiD− and DiD+ populations indicated that DiD+ cells in both MCF-7 and MDA-MB-231 cell lines were enriched for cells in the G_2_/M-phase of the cell cycle. A number of instances of label-retaining cells having an increased G_2_/M-fraction compared to their rapidly proliferating counterparts have been reported in the existing literature across various cancer types (breast, prostate, intestinal, myeloid cell, brain and ovarian) [7, 10, 11]. It has also previously been reported that such G_2_/M-phase arrest is associated with multi-drug chemoresistance and a propensity to evade apoptosis [32, 33]. The latter observation implies that, while reduced mitotic activity itself is likely to contribute to the survival of label-retaining cells in response to chemotherapy, it may not be the sole means by which de novo drug resistance occurs; growth arrest or slowed cell cycle transition could effectively increase the time for drug efflux, drug metabolism or repair of drug-induced cellular stress, and thereby enable evasion of pro-apoptotic signals in the relatively quiescent cell fraction. Indeed, this may have been a significant contributory factor in the enhanced survival and enrichment of DiD+ cells in MCF-7 and MDA-MB-231 cultures treated with anti-neoplastic drugs. In support of this hypothesis, the expression of anti-apoptotic proteins has frequently been observed in quiescent normal and cancer stem cell populations and has been shown to contribute to their enhanced survival following chemotherapy [34]. Increased DNA damage repair pathway activation, up-regulation of xenobiotic drug pumps, and elevated enzymatic drug metabolism (particularly by ALDH) have also been reported in quiescent stem cell populations [35, 36], and we similarly found ALDH activity to be significantly up-regulated within the slow-cycling, chemotherapy-resistant DiD+ fraction of both MCF-7 and MDA-MB-231 cell lines.

Given the slow-cycling and therapy-resistant nature of the DiD+ cell fraction, it appears that there could be an associated between slow-cycling cells and the purported cancer stem cell population. A number of studies across various cancer types have transitively linked quiescence to the cancer stem cell phenotype through retrospective functional validation of putative cancer stem cell populations isolated using cell surface markers [12, 20, 37]. In contrast, studies in which the prospective identification of a quiescent cell population was undertaken through label retention assays have demonstrated only partial overlap with cell surface marker signatures associated with the supposed cancer-type-specific stem cell population [7, 8]. Our findings are in agreement with the latter reports; we observed only a modest increase in ALDH activity differentiated the DiD− and DiD+ populations, and DiD+ cells were not enriched for the CD44^+^CD24^−/low^ putative breast CSC marker signature. Notably, the DiD+ sub-population in both MCF-7 and MDA-MB-231 cell lines did show a significant enrichment with cells expressing the CD44^+^CD24^+^ phenotype. The functional implications of enrichment for CD44^+^CD24^+^ cells in the DiD+ population and the nature of other cell sub-populations (e.g. DiD+ ALDH^−^) remain to be established. While the CD44^+^CD24^−/low^ surface marker signature and high ALDH activity have been widely used to identify the putative CSC population since the pioneering studies of Al-Hajj et al. [26] and Ginestier et al. [24], respectively, more recent studies have demonstrated that the CD44^+^CD24^−/low^ and ALDH^+^ marker profiles associated with putative breast CSCs identify minimally overlapping, spatiotemporally distinct populations across different breast cancer sub-types [38]. These studies exemplify that the concept of exclusive tumour cell sub-sets possessing increased nascent capacity for tumour propagation is still evolving. Another very notable example of this situation in breast cancer is that of the MDA-MB-231 cell line; data reported here and by others has shown that in excess of 99% of the total cell population express the CD44^+^CD24^−/low^ marker signature that is supposed to describe highly tumourigenic breast CSC, yet only a minority fraction of the cell population possesses the ability to initiate population re-growth [39]. Indeed, an often overlooked finding of the landmark study undertaken by Al-Hajj et al. [26] was that CD44^+^CD24^+^ breast cancer populations also remained viable and exhibited tumourigenicity in xenotransplantation studies but seemingly possessed reduced proliferative capacity compared to the CD44^+^CD24^−/low^ cells that were taken to represent the stem-like fraction. Based on these findings it seems plausible that the CD44^+^CD24^−/low^ signature was implicitly linked to enhanced tumourigenicity simply due to the rapid expansion of this population coupled with an insufficient follow-up period in mice injected with CD44^+^CD24^+^ cells, leading to the conclusion that the former exclusively possessed tumourigenic potential. In support of this, a number of other studies have indicated that stem cell activity in breast cancer is not exclusively limited to the CD44^+^CD24^−/low^ phenotype but that CD44^+^CD24^+^ can be equally tumourigenic in mouse xenograft models, most notably illustrated by Meyer et al. [40] in oestrogen receptor-negative disease. Moreover, evidence from a more recent study indicates that CD24 status has little bearing on tumourigenic potential in breast cancer and that CD24^−/low^ status can in fact reduce tumour initiation in some murine models [41]. Given the potential for a majority of cancer cells or numerous intrinsic sub-populations to display tumourigenicity, we believe that the identification of a slow-cycling and inherently therapy resistant cellular sub-set capable of leading to tumor recurrence is both biologically and clinically significant, independent of a cancer stem cell model.

The use of Vybrant^®^ DiD to identify slow-cycling cancer cell populations is a simple and highly reproducible process that yields an easily identifiable population for isolation and further characterisation. In addition, the use of Vybrant^®^ DiD and analogous dyes offers the additional distinct advantage over other pulse-chase techniques (e.g. BrdU or EdU) of allowing live cells to be isolated, allowing functional studies to be carried out and comparisons to be made between the different populations. One drawback of this method is that sufficient time in culture (several weeks) must elapse before label-retaining cells can be identified. This factor has a significant knock-on effect on it’s prospective utility for primary cultures, in that passaging this material multiple times could potentially alter the characteristics from those of the original tissue. In addition, the capability of the dye-retaining, therapy-resistant cell population to form new tumours can ultimately only be demonstrated by implantation in vivo. On balance, however, this method does offer the possibility of obtaining much more material for characterisation when compared to traditional means of identifying prospective stem-like cells, and the progressive dilution or retention of dye is reliant on a functional cellular phenotype that is independent of the expression of protein markers with poorly understood functional roles in mitotic dynamics.

In summary, the work reported here details a highly reproducible and user-friendly method to enrich, isolate and characterise a live population of chemotherapy-resistant tumour cells based on a functional, mitotically quiescent phenotype. Further characterisation of this population could reveal biological programmes associated with mitotic quiescence and de novo drug resistance and thereby yield novel targets for therapies enabling elimination of the cells responsible for breast cancer recurrence.

## Electronic supplementary material

Below is the link to the electronic supplementary material.

Supplementary material 1 Supplementary Fig. 1 Flow Cytometric Analysis of Vybrant^®^ DiD Retention. (a) Cellular debris, identified by low particle size or forward-scatter (FSC) and granularity or side-scatter (SSC), was first gated-out of the event population. (b) The resultant population was then divided into single cell and non-single cell events. (c) Single cell events were further divided into live and dead cell populations based on fluorescence intensity of viability dye (propidium iodide) staining relative to autofluorescence of an unlabelled cell sample analysed at the same detector channel voltage. (d) Live, single cells were then divided into Vybrant^®^ DiD-negative (DiD-) or Vybrant^®^ DiD-positive (DiD+) (TIFF 3068 KB)

Supplementary material 2 Supplementary Fig. 2 Chemotherapy Dose-Response Curves. (a) Dose-response curve for the MCF-7 cell line established following a 72-hour period of exposure to 1pM – 100 µM doxorubicin. (b) Dose-response curve for the MCF-7 cell line established following a 72-hour period of exposure to 10fM – 1 µM paclitaxel. (c) Dose-response curve for the MDA-MB-231 cell line established following a 72-hour period of exposure to 1pM – 100 µM doxorubicin. (d) Dose-response curve for the MDA-MB-231 cell line established following a 72-hour period of exposure to 10fM – 1 µM paclitaxel. Cell survival at each drug concentration was established using the MTT assay and is expressed as a percentage of Abs_570nm_ recorded for samples exposed to the respective vehicle control solution. Data are expressed as the mean ± SEM (TIFF 2937 KB)

Supplementary material 3 Supplementary Fig. 3. Vybrant^®^ DiD for Long-Term Lineage Tracing In Vitro. (a) The percentage of positively-stained MCF-7 and MDA-MB-231 cells immediately after labelling of cultures with Vybrant^®^ DiD (n = 3). Representative images of adherent MCF-7 and MDA-MB-231 cells at 4 hours post-staining with DiD fluorescence (red) are also shown (scale bar = 100 µm). (b) The percentage of viable cells in Vybrant^®^ DiD-stained MCF-7 and MDA-MB-231 cultures (final concentration of DiD = 5 µM) compared to control cultures not exposed to Vybrant^®^ DiD (n = 3, unpaired t-test, ns = not significant or P > 0.05). (c) Proliferation curves for Vybrant^®^ DiD-stained MCF-7 and MDA-MB-231 cultures compared to control cultures not exposed to Vybrant^®^ DiD (n = 3, two-way ANOVA with Sidak’s multiple comparison, ns = not significant or P > 0.05). (d) Correlation of the number of cells in Vybrant^®^ DiD-stained MCF-7 and MDA-MB-231 cultures with the mean fluorescence intensity of Vybrant^®^ DiD staining after one passage (4 days) of culture growth (n = 3). All graphical data are expressed as mean ± SEM (TIFF 6612 KB)

Supplementary material 4 (PDF 44 KB)

## Abbreviations

BMP1: Bone morphogenetic protein 1
BrdU: Bromodeoxyuridine (5-bromo-2’-deoxyuridine)
CD24: Cluster of differentiation 24
CD44: Cluster of differentiation 44 (hyaluronan receptor)
CDH1: Cadherin 1, type 1, E-cadherin (epithelial)
CDH2: Cadherin 2, type 1, N-cadherin (neuronal)
CSC: Cancer stem cell
CTNNB1: Catenin (cadherin-associated protein), beta 1
DEAB: Diethylaminobenzaldehyde (4-Dimethylaminobenzaldehyde)
DiD-: Vybrant^®^ DiD-negative (non-label-retaining), fast-cycling cell
DiD+: Vybrant^®^ DiD-positive (label-retaining), slow-cycling cell
EdU: Ethynyldeoxyuridine (5-ethynyl-2’-deoxyuridine)
EGFR: Epidermal growth factor receptor
EMT: Epithelial-to-mesenchymal transition
FZD7: Frizzled family receptor 7
IC_95_: 95% Inhibitory concentration
JAG1: Jagged 1
MET: Mesenchymal-to-epithelial transition
NOTCH1: Notch 1
SMAD2: SMAD family member 2
SNAI2: Snail homolog 2
TGFB1: Transforming growth factor, beta 1
TGFB2: Transforming growth factor, beta 2
TGFB3: Transforming growth factor, beta 3
WNT5B: Wingless-type MMTV integration site family, member 5B
VIM: Vimentin
