# Supplementary figures and images for: Chemotherapy resistance and stemness in mitotically quiescent human breast cancer cells identified by fluorescent dye retention
Source: Clin Exp Metastasis. 2018 Oct 30;35(8):831–46. doi: 10.1007/s10585-018-9946-2 (PMC6267670; doi:10.1007/s10585-018-9946-2)

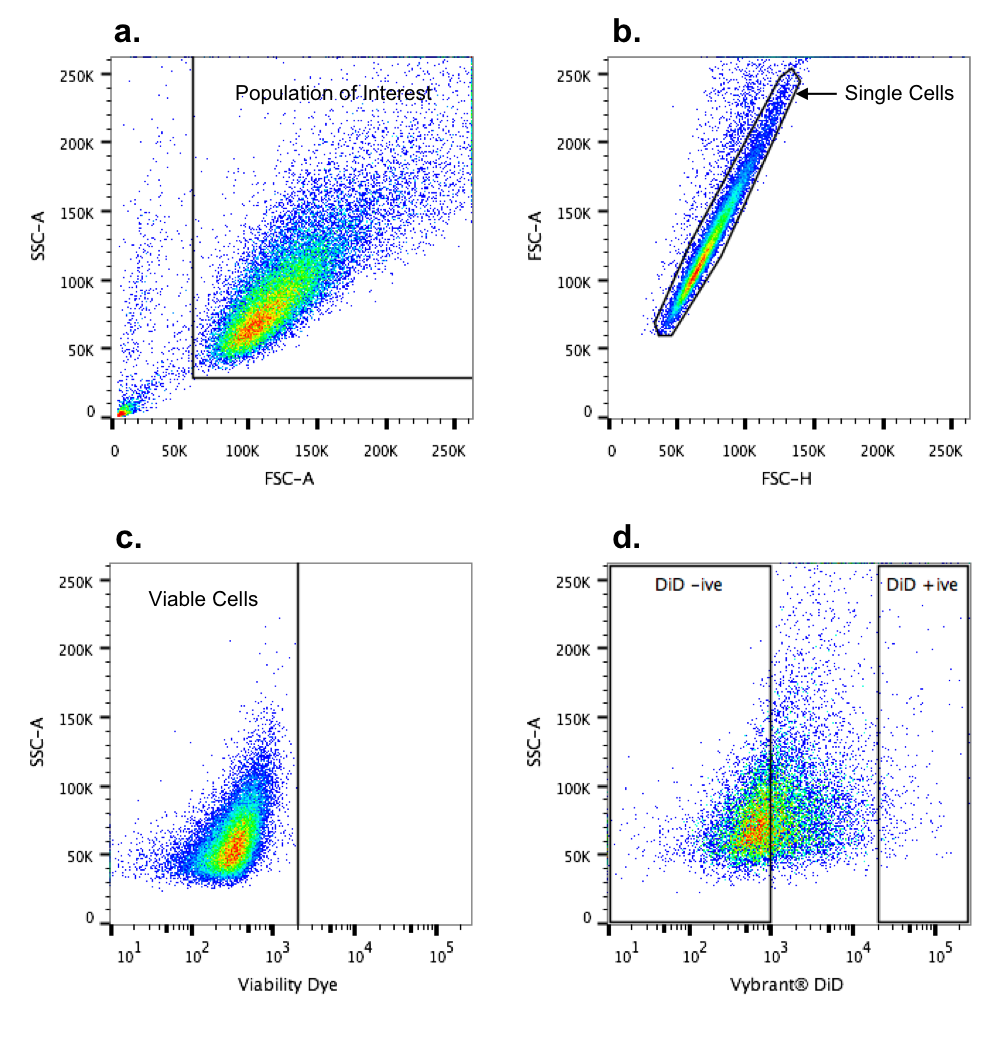

Supplement: Supplementary file 1 — Supplementary material 1 Supplementary Fig. 1 Flow Cytometric Analysis of Vybrant® DiD Retention. (a) Cellular debris, identified by low particle size or forward-scatter (FSC) and granularity or side-scatter (SSC), was first gated-out of the event population. (b) The resultant population was then divided into single cell and non-single cell events. (c) Single cell events were further divided into live and dead cell populations based on fluorescence intensity of viability dye (propidium iodide) staining relative to autofluorescence of an unlabelled cell sample analysed at the same detector channel voltage. (d) Live, single cells were then divided into Vybrant® DiD-negative (DiD-) or Vybrant® DiD-positive (DiD+) (TIFF 3068 KB) [file 10585_2018_9946_MOESM1_ESM.tiff]
