# Supplementary material for: Chemotherapy resistance and stemness in mitotically quiescent human breast cancer cells identified by fluorescent dye retention
Source: Clin Exp Metastasis. 2018 Oct 30;35(8):831–46. doi: 10.1007/s10585-018-9946-2 (PMC6267670; doi:10.1007/s10585-018-9946-2)
Supplement: Supplementary file 4 — Supplementary material 4 (PDF 44 KB) [file 10585_2018_9946_MOESM4_ESM.pdf]

Supplementary Table 1. Gene Assays in the Qiagen Custom RT^2 Profiler PCR Array

Gene Symbol	Ref Seq No.	Description	Catalogue Number
BMP1	NM_006129	Bone morphogenetic protein 1	PPH00515B
CDH1	NM_004360	Cadherin 1, type 1, E-cadherin (epithelial)	PPH00135F
CDH2	NM_001792	Cadherin 2, type 1, N-cadherin (neuronal)	PPH00636F
CTNNB1	NM_001904	Catenin (cadherin-associated protein), beta 1, 88kDa	PPH00643F
EGFR	NM_005228	Epidermal growth factor receptor	PPH00138B
FZD7	NM_003507	Frizzled family receptor 7	PPH02420B
JAG1	NM_000214	Jagged 1	PPH06022B
NOTCH1	NM_017617	Notch 1	PPH00526C
SMAD2	NM_005901	SMAD family member 2	PPH01949F
SNAI2	NM_003068	Snail homolog 2 (Drosophila)	PPH02475A
TGFB1	NM_000660	Transforming growth factor, beta 1	PPH00508A
TGFB2	NM_003238	Transforming growth factor, beta 2	PPH00524B
TGFB3	NM_003239	Transforming growth factor, beta 3	PPH00531F
VIM	NM_003380	Vimentin	PPH00417F
WNT5B	NM_032642	Wingless-type MMTV integration site family, member 5B	PPH02447C
ACTB	NM_001101	Actin, beta	PPH00073G
B2M	NM_004048	Beta-2-microglobulin	PPH01094E
GAPDH	NM_002046	Glyceraldehyde-3-phosphate dehydrogenase	PPH00150F
HPRT1	NM_000194	Hypoxanthine phosphoribosyltransferase 1	PPH01018C
RPLP0	NM_001002	Ribosomal protein, large, P0	PPH21138F
HGDC	SA_00105	Human Genomic DNA Contamination Control	PA-031
RTC	SA_00104	Reverse Transcription Control	PPX63340
PPC	SA_00103	Positive PCR Control	PPX63339
